# Clinical Outcomes of Cardiac Transplantation in Heart Failure Patients with Previous Mechanical Cardiocirculatory Support

**DOI:** 10.3390/jcm14010275

**Published:** 2025-01-06

**Authors:** Michele D’Alonzo, Amedeo Terzi, Massimo Baudo, Mauro Ronzoni, Nicola Uricchio, Claudio Muneretto, Lorenzo Di Bacco

**Affiliations:** 1 Cardiac Surgery Unit, Spedali Civili, University of Brescia, 25124 Brescia, Italy; m.ronzoni001@unibs.it (M.R.); cmuneretto@unibs.it (C.M.); lorenzo.dibacco@hotmail.it (L.D.B.); 2 Cardiac Surgery Unit, ASST Papa Giovanni XXIII, 24127 Bergamo, Italy; aterzi@asst-pg23.it (A.T.); nuricchio@asst-pg23.it (N.U.); 3 Department of Cardiac Surgery Research, Lankenau Institute for Medical Research, Main Line Health, Wynnewood, PA 19096, USA; massimo.baudo@icloud.com

**Keywords:** ventricular assist device, bridge to transplant, heart failure, heart transplantation

## Abstract

**Objectives:** Heart failure (HF) remains a significant public health issue, with heart transplantation (HT) being the gold standard treatment for end-stage HF. The increasing use of mechanical circulatory support, particularly left ventricular assist devices (LVADs), as a bridge to transplant (BTT), presents new perspectives for increasingly complex clinical scenarios. This study aimed to compare long-term clinical outcomes in patients in heart failure with reduced ejection fraction (HFrEF) receiving an LVAD as BTT to those undergoing direct-to-transplant (DTT) without mechanical support, focusing on survival and post-transplant complications. **Methods:** A retrospective, single-center study included 105 patients who underwent HT from 2010. Patients were divided into two groups: BTT (n = 28) and DTT (n = 77). Primary endpoints included overall survival at 1 and 7 years post-HT. Secondary outcomes involved late complications, including organ rejection, renal failure, cardiac allograft vasculopathy (CAV), and cerebrovascular events. **Results:** At HT, the use of LVADs results in longer cardiopulmonary bypass and cross-clamping times in the BTT group; nevertheless, surgical complexity does not affect 30-day mortality. Survival at 1 year was 89.3% for BTT and 85.7% for DTT (*p* = 0.745), while at 7 years, it was 80.8% and 77.1%, respectively (*p* = 0.840). No significant differences were observed in the incidence of major complications, including permanent dialysis, organ rejection, and CAV. However, a higher incidence of cerebrovascular events was noted in the BTT group (10.7% vs. 2.6%). **Conclusions:** LVAD use as BTT does not negatively impact early post-transplant survival compared to DTT. At long-term follow-up, clinical outcomes remained similar across groups, supporting LVADs as a viable option for bridging patients to transplant.

## 1. Introduction

The prevalence of heart failure (HF) is steadily rising in both genders, with a particular increase observed in the elderly cohorts of populations, a trend in part attributable to advancements in medical treatment and improved survival rates [1]. For patients with end-stage HF, refractory to medical treatment, heart transplantation (HT) remains to date the gold standard of care. Nevertheless, the limited availability of donor organs has resulted in a significant increase in the utilization and development of mechanical circulatory support (MCS), such as left ventricular assist devices (LVADs), as a bridge to transplant (BTT) or bridge to candidacy (BTC) [2]. The widespread use of LVADs as a bridge to heart transplantation has improved survival rates for patients awaiting donor organs and has also enabled many candidates to endure extended waiting periods [3,4].

Although the therapeutic benefits of LVAD implantation are well documented [5], they are also associated with several complications, including gastrointestinal bleeding, neurological events, infection, pump thrombosis, and right ventricular failure [6,7]. Recent studies have also highlighted some negative effects of long-term LVADs on organ function, in particular, regarding liver [8] and renal dysfunction [9]. This has brought back into focus a highly controversial topic. It is therefore pivotal to better understand the long-term impact of patients who receive an LVAD as a bridge to HT. Early reports, particularly those that examined the earlier generations of LVADs, suggested that the use of LVADs could adversely affect survival following transplantation [10,11].

However, in the present day, survival outcomes of heart transplantation from durable mechanical circulatory support (MCS) using the latest devices (HeartMate III) are promising [5,12]. A literature review examined 428 papers on this subject, concluding that decreased survival was more likely in patients suffering from dilated cardiomyopathy, patients transplanted within two weeks of LVAD implantation, or patients who underwent BTT prior to 2003 [13]. Indeed, post-transplant outcomes in patients bridged to transplant with temporary mechanical circulatory support devices, like extracorporeal membrane oxygenation, were associated with a higher risk of mortality [14,15]. While the mechanisms underlying these outcomes remain complex, one established finding is that the duration of the LVAD does not have a direct effect on post-transplant survival [16].

More than a decade later, it has become essential to reassess these findings with a broader perspective: advances in technology, including smaller devices, longer battery life, and an improved understanding of the pathophysiology of circulation in patients with LVADs, reinforce the importance of these devices in the management of advanced heart failure without compromising long-term post-transplant outcomes.

This study aims to provide an overview of the long-term outcomes in patients in heart failure with reduced ejection fraction (HFrEF) receiving an LVAD as a bridge to heart transplantation compared to those receiving heart transplantation with medical therapy alone, and to offer insights into the various complications associated with pre-transplant mechanical support.

## 2. Materials and Methods

### 2.1. Ethical Statement

A retrospective, observational, single-center study was conducted analyzing clinical outcomes of 105 patients who underwent cardiac transplantation between January 2010 and June 2020 at Hospital “Papa Giovanni XIII” in Bergamo (Italy). Patients gave consent for surgical intervention and for data publication. Ethical review and approval were waived for this study due to the observational and retrospective nature of the study.

### 2.2. Patient Population

Adult patients (aged ≥ 18 years) with ischemic or dilated cardiomyopathy who underwent isolated heart transplantation were included in this study.

Patients with a left ventricular ejection fraction (LVEF) greater than 40% were excluded from the study, limiting the analysis to patients with heart failure with reduced ejection fraction (HFrEF). Patients with heart failure with mid-range ejection fraction (HFmrEF) or preserved ejection fraction (HFpEF) were not considered.

Exclusion criteria were congenital disease, other types of cardiomyopathy, and patients who died before heart transplantation. We analyzed patients who received an LVAD, either as a bridge to transplant (BTT) or bridge to candidacy (BTC), collectively categorized under the BTT group. The analysis included the following devices: HeartMate II (Thoratec Corporation; Pleasanton, CA, USA), HeartWare (Medtronic; Minneapolis, MN, USA), and HeartMate III (Abbott Laboratories; Chicago, IL, USA). Patients in this latter group were compared to those undergoing de novo heart transplantation (direct-to-transplant, DTT). Patients supported with extracorporeal LVADs, right ventricular assist devices, biventricular assist devices, and total artificial hearts were excluded.

### 2.3. Endpoints and Definitions

Follow-up was conducted up to January 2022 (mean follow-up was: 5.76 ± 3.8 years). The primary outcome of interest was overall survival at 1 and at 7 years. The secondary outcomes included the occurrence of adverse events during follow-up. Adverse events were defined as significant arrhythmias, cerebrovascular events (excluding those occurring before or during heart transplantation), renal failure (eGFR < 30 mL/min) or the need for permanent dialysis, organ rejection, and cardiac allograft vasculopathy (CAV), according to relative International Society for Heart and Lung Transplantation (ISHLT) classifications [17,18].

### 2.4. Data Collection and Follow-Up Protocols

Patients’ clinical data and parameters were evaluated at four specific time-points:

– Baseline before HT: physical characteristics, assessment of clinical, history, heart failure etiology, echocardiographic, hemodynamic, and biochemical data of patients at the time of heart transplantation.
– LVAD-related complications between device implantation and heart transplantation (only for BTT group).
– Surgical data, early mortality (at 30-day), and morbidity during hospitalization, for heart transplantation.
– Evaluation of transplant-related complications by phone interview or direct clinical examination, at the last FU.

All patients underwent HT with bicaval anastomosis. All patients received anti-rejection therapy with calcineurin inhibitors, cyclosporine or tacrolimus, mycophenolate mofetil, and prednisone according to the hospital protocols. Endo-myocardial biopsies (EMBs) were performed at 1 year, to determine the occurrence of rejection, according to hospital protocols (one biopsy every week in the first month, one biopsy every two weeks until the third month, one biopsy every month until the sixth month, and one biopsy every two months until the twelfth month; after one year, no routine EMBs were performed if no clinical suspicion of rejection was present): biopsy classification followed the revised International Society for Heart and Lung Transplantation (ISHLT) staging classification [17].

To assess the presence of CAV, coronarography was scheduled at 3 and 5 years after transplantation, then every 5 years or following clinical suspicion. The grade of CAV was assessed using the ISHLT classification [18].

### 2.5. Statistical Analysis

The distribution of variables was evaluated using the Kolmogorov–Smirnov 1-sample test. Continuous variables were presented as mean ± standard deviation (SD) for variables with a normal distribution or as median (1st and 3rd interquartile) for data without Gaussian distribution. Categorical variables were expressed as absolute number of frequency (percentages). Independent t-tests were used to compare normally distributed data. For data without normal distribution, the Mann–Whitney test for unpaired continuous variables was used. The chi-square test was used to compare categorical data, and Fisher’s exact test was used when the minimum cell size requirements for the chi-square test were not met. Kaplan–Meier estimates were used to assess long-term post-transplantation survival. Microsoft Office Excel (Microsoft, Redmond, WA, USA) was used for data extraction, and all analyses were performed in R, version 4.3.1 (R Software for Statistical Computing, Vienna, Austria) within RStudio. The R packages used were “survival”, “survminer”, “dplyr”, and “ggplot2”.

## 3. Results

Seventy-seven (n = 77) patients were in the DTT group, while twenty-eight patients were in the BTT group. Table 1 shows the baseline differences between the two groups. The BTT group had a higher BMI (26.4 ± 5.0 kg/m^2^ vs. 23.1 ± 3.9, *p* < 0.05) with a lower percentage of patients with atrial fibrillation (n = 1, 3.6% vs. 20, 26.0%, *p* = 0.004). General comorbidities, such as liver and kidney function status, were comparable between the groups. The etiology of heart failure was balanced, with 50% of each group affected by ischemic heart disease and the other 50% by dilated cardiomyopathy (*p* = 0.953).

### 3.1. LVAD Population

The BTT population included 28 patients (mean age: 48.5 ± 11.4 years). Considering the broad recruitment period of the study (January 2010 to June 2020) and the availability of devices, 21 patients (75%) received a HeartWare implant, which was the most used LVAD, 3 patients (10.7%) received a HeartMate II, and 4 patients (14.3%) received a HeartMate III. The bridge-to-transplant duration was 554 ± 346 days, during which the complications outlined in Table 2 were recorded. It was not possible to trace patients who had LVAD implantation but were not transplanted for various reasons (death, refusal to transplant, transplantation to another hospital center, off transplant list).

### 3.2. Heart Transplantation Hospitalization

Data at the time of hospitalization for heart transplantation are depicted in Table 3. Aortic cross-clamp and cardiopulmonary bypass times were longer in the BTT group, without impact on in-hospital mortality between the two groups, BTT: one patient (3.6%) vs. DTT: four patients (5.2%) (*p* = 0.999).

A higher percentage of perioperative cerebrovascular events was observed in the BTT group: three patients, 10.7% vs. DTT: one patient, 1.3%, *p* = 0.057). No significant differences were observed in the incidence of various complications: acute organ rejection (*p*= 0.723), bleeding requiring surgery (*p* = 0.442), dialysis (*p* = 0.702), or ECMO (*p* = 0.717).

### 3.3. Primary Endpoint

**Figure 1:**
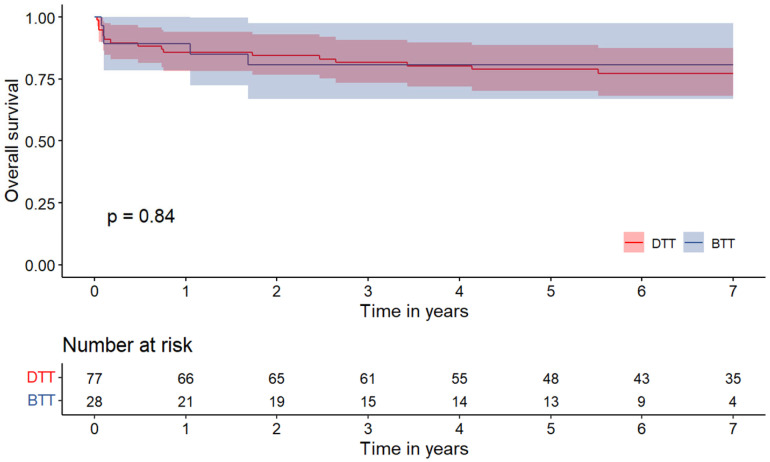
Kaplan–Meier curves for all-cause mortality in patients who underwent heart transplantation. DTT: direct-to-transplantation; BTT: bridge to transplantation.

Survival at 1 and at 7 years is shown in Figure 1. Of the 105 patients undergoing HT, 101 (96.2%) patients completed at 1-year FU. When stratified by surgical strategy for HT, there was no significant difference in terms of 1-year survival (BTT 89.3 ± 11.4% vs. DTT 85.7± 7.8%; *p* = 0.745). At 7-year follow-up, the survival rate was 80.8 ± 15.3% for the BTT group and 77.1 ± 9.6% for the DTT group (*p* = 0.840).

### 3.4. Secondary Endpoints

The secondary outcomes during follow-up are depicted in Table 4. When considering cumulative arrhythmias (permanent atrial fibrillation, permanent pacemaker, or defibrillator), no significant differences were observed between the two groups (*p* = 0.999). Similarly, the incidence of cardiac allograft vasculopathy was comparable, with 14.2% in the BTT group and 18.2% in the DTT group (*p* = 0.775). A trend toward a higher incidence of postoperative cerebrovascular events was observed in the BTT group (10.7% vs. 2.6%, *p* = 0.117), while there was a greater need for dialysis in the DTT group (14.3% vs. 3.6%, *p* = 0.175). Regarding overall cardiac organ rejection, approximately one-quarter of patients in each group experienced this adverse event, with no statistically significant difference (*p* = 0.644); even when considering the more severe grades (grade IIIA and grade IIIB), no statistically significant difference was found.

## 4. Discussion

This study presents an updated analysis of long-term outcomes in patients in heart failure with reduced ejection fraction (HFrEF) undergoing heart transplantation after receiving mechanical circulatory support with a left ventricular assist device (LVAD). The main findings can be summarized as follows:

(I) At the time of heart transplantation, the use of an LVAD introduces technical challenges that result in longer cardiopulmonary bypass and cross-clamping times in the BTT (bridge to transplant) group; nevertheless, the increase in surgical complexity does not affect 30-day mortality.
(II) The overall survival at 1 year was comparable between the two groups, with no significant difference between the BTT and DTT (direct-to-transplant) patients.
(III) At 7 years, survival rates and comorbidities, including post-transplant complications such as cardiac allograft vasculopathy (CAV) and organ rejection, were similar between both groups.
(IV) Adverse neurological events (composite of TIA/stroke) occurred in 39.6% of patients during the BTT phase, indicating a concern on neurological risk mechanical support.

Heart transplantation is considered the gold standard treatment for patients with advanced heart failure [2]. However, nowadays, there is a trend toward the increasing use of LVADs as a bridge-to-transplant (BTT) or bridge-to-candidacy (BTC) solution. This phenomenon is mostly attributable to the increased number of patients with non-persistent contraindications to HT and the scarcity of heart organ donors [19,20]. Mechanical circulatory support gives patients a chance to reach the state of candidacy or transplant, but the surgical implications related to LVAD implantation should not be overlooked. Heart transplant surgical time tends to be longer for those patients, because of adhesions of previous cardiac surgery and LVAD removal, making surgery more technically demanding. The present study showed that the average aortic cross-clamp (ACC) and cardiopulmonary bypass (CPB) time were, respectively, 34.6 and 71.5 min shorter in the DTT group compared to the BTT group. Although the ACC and CPB duration are depicted from the literature as predictors of adverse outcomes, particularly in elective and low-risk procedures, we found no significant difference in early mortality between the two groups despite the significant difference in CPB and ACC times (hospital mortality: 3.6% BTT vs. 5.2% DTT, *p*: ns). The reported results in the BTT patients are consistent with the results of Fukuhara and colleagues in a cohort of patients with a similar bridge duration [21] with early mortality found to be slightly lower compared to the mortality rates in similar patient subsets reported in other studies, which range from 5% to 10% [15,22]. This finding could be explained by the hemodynamic support provided by LVADs prior to transplantation conferring a higher degree of clinical stability that can mitigate the potential negative impact of longer procedural times. Moreover, we found a 30-day mortality in the BTT group slightly lower than the previous literature in a similar subset of patients ranging from 5 to 10% [15,22]. In this series, the wide use of post-transplant support with ECMO (25% in BTT group and 28.6% in DTT group) might also have contributed to enhancing patients’ survival in both groups. Post-transplant ECMO is typically used when the newly transplanted heart struggles to maintain adequate cardiac output due to primary graft dysfunction, rejection, or other complications such as severe pulmonary hypertension. The use of ECMO to unload the heart allowed the transplanted organ and lung to recover [23]. In addition, in this study, 89.3% of patients in the BTT group received a third-generation LVAD, namely HeartWare and HeartMate III, that can provide more benefits and less harm to patients who are waiting for HT [24].

Moreover, 1- and 7-year survival rates were also found to be comparable between the two groups (1-year: BTT 89.3 ± 11.4% vs. DTT 85.7± 7.8%; 7-year: BTT 80.8 ± 15.3% vs. DTT 77.1 ± 9.6% (*p* = 0.840), supporting the conclusion that the use of LVADs as a bridge-to-transplant strategy does not adversely affect early post-transplant survival.

These findings are consistent with the results reported in a French institution by Petroni et al. [25]. Conversely, the same differ from those of Truby et al’s. [26] on an American population, where patients who had an LVAD as a bridge to HT experienced higher mortality within the first five years post-transplant. Although the primary outcome of the aforementioned papers is the same, it is crucial to consider the significant differences between the populations considered: Truby’s and Petroni’s studies included different ethnic groups, while our analysis exclusively involved Caucasian patients; racial differences in the population enrolled may represent an important risk factor for patients’ survival. Furthermore, the etiology of heart failure varied considerably across these studies. In our cohort, there was a balance between ischemic and dilated cardiomyopathy, whereas in Petroni’s study, ischemic cardiomyopathy accounted for only 32% of cases while Truby’s analysis also included congenital heart diseases among the etiologies considered.

A further notable finding in the present study is that no significant differences were observed between the two groups in terms of major complications such as stroke, organ rejection, CAV, and chronic kidney failure. It is noteworthy, as previous authors have highlighted the risk of humoral sensitization in patients with LVAD implantation [12,27], suggesting that they may be more susceptible to graft rejection. In this regard, Arnaoutakis et al. identified a correlation between LVAD use and an increased rate of primary graft dysfunction, yet found no adverse effects on survival [28]. In our study, there was no significant difference in the incidence of any grade of CAV, nor in organ rejection. Petroni’s analysis also reported a low morbidity rate among patients who underwent bridge to HT, compared to those who received only medical therapy [25]. This protective effect was attributed to improved systemic perfusion, which helped restore hemodynamic- and reverse kidney-related metabolic and cellular damage prior to HT, thereby also lowering the risk of post-transplant morbidity. The presented data support these findings, as demonstrated by the lower incidence of permanent dialysis (BTT 3.6% vs. DTT 14.3%) and higher eGFR values (Table 4) observed in patients who underwent LVAD implantation.

Despite several studies that attempted to investigate the short- and long-term clinical outcomes of patients treated with LVADs and those who undergo transplantation without bridging, the superiority of BTT over DTT is to date uncertain. The main reason lies in the absence of randomized trials due to the significant complexity of the patients and the limited availability of organs. Moreover, in retrospective registries and analysis, the populations compared are often highly heterogeneous as previously described.

Finally, the drawbacks related to the use of LVADs must be considered. The LVAD-related complications reported in the present study are comparable with the previous literature [29,30,31], while a higher incidence of neurological events during the BTT phase was reported when compared to previous experiences (39.6% in this analysis versus 8–25% [32,33]). This difference may be related to two factors. Firstly, most studies consider only major strokes (both ischemic and hemorrhagic) while in the present research, TIAs were also included. An additional consideration is that most of the latest studies primarily examine the outcomes of HeartMate devices within the first year, whereas our population had a higher prevalence of HeartWare devices. Notably, the HeartWare device was withdrawn from the market in 2021 due to an increased risk of neurological events and mortality compared to other devices. Moreover, as suggested by Fukuhara, the advantage of mechanical support with an LVAD gives the best results when the bridge period is shorter than 2 years; after this limit, BTT is associated with worse results in terms of survival after HT when compared to DTT and a shorter BTT period [21].

*Strengths and Study Limitations*

Only patients treated after 2010 were enrolled in this study. In our center, a post-transplant ECMO support protocol was routinely available if necessary as well as the newest anti-rejection protocol and third-generation LVAD devices, and the results of this study align with the results of larger studies on this topic. Furthermore, follow-up was completed in 96.2% of patients at one year, with very few patients lost at longer FU. Nevertheless, the current study has several limitations. It is a retrospective and single-center study; the small sample size was not eligible for multivariate analysis to identify primary endpoint predictive factors. Since this study was not randomized, assessing the superiority of one management over the other was not possible. Therefore, the adjustment of baseline characteristics, which is essential for accurate comparison among groups, was not possible, and consequently, we did not perform advanced statistical analyses such as multivariate analysis. Furthermore, despite the use of different types of LVADs, the results were mainly related to HeartWare implantation (75% of BTT patients). Lastly, we acknowledge as a limitation of this study the exclusion of etiologies other than ischemic and dilated cardiomyopathy.

## 5. Conclusions

Despite the progress in MCS support, heart transplantation remains the gold standard therapy for patients in heart failure with reduced ejection fraction (HFrEF). The one-year mortality post-transplantation was 13% (10.7% in BTT and 14.3% in DTT), in line with other European studies. Therefore, LVAD implantation as BTT does not increase the risk of mortality following heart transplant, as compared with patients bridged with medical therapy.

## Figures and Tables

**Table 1:** Baseline characteristics before heart transplantation.

	BTT (n = 28)	DTT (n = 77)	*p* Value
Age, years	54 (43–58)	53 (42–49)	0.954
Gender, male	25 (89.3)	65 (84.4)	0.528
BMI, Kg/m^2^	26.4 ± 5.0	23.1 ± 3.9	0.001
Etiology			0.953
ICM	14 (50.0)	39 (50.6)	
DCM	14 (50.0)	38 (49.4)	
Atrial fibrillation	1 (3.6)	20 (26.0)	0.004
Chronic dialysis	1 (3.6)	2 (2.6)	0.999
Hemodynamic status			
LVEF, %	24 (20–26)	20 (18–25)	0.137
sPAP, mmHg	41.8 ± 18.4	40.2 ± 15.4	0.686
Wedge, mmHg	18.5 ± 8.8	19.8 ± 9.3	0.561
CO, L	4.1 ± 1.0	3.5 ± 1.1	0.031
CI, L/m^2^	2.0 (1.8–2.6)	1.9 (1.6–2.1)	0.128
PVR, WU	2.2 (1.4–2.9)	2.4 (1.5–3.3)	0.291
Laboratory			
Creatinine, mg/dL	1.1 ± 0.3	1.2 ± 0.4	0.392
Total bilirubin, mg/dL	1.1 ± 0.8	1.4 ± 1.8	0.349

BTT: bridge to transplant; CI: cardiac index; DCM: dilatative cardiomyopathy; ICM: ischemic cardiomyopathy; CO: cardiac output; DTT: direct-to-transplant; LVEF: left ventricular ejection fraction; PVR: pulmonary vascular resistance; sPAP: systolic pulmonary artery pression.

**Table 2:** BTT details.

	BTT (n = 28)
Age at LVAD implant, years	48.5 ± 11.4
Implantable device	
HeartMate II	3 (10.7)
HeartMate III	4 (14.3)
HeartWare	21 (75.0)
Period of bridge, days (mean ± SD)	554 ± 346
Period of bridge, days (range)	(26–1559)
Complications	
Readmission for HF, n (%)	5 (17.9)
GI bleeding, n (%)	8 (28.6)
Cerebrovascular events, n (%)	11 (39.3)
Arrhythmias, n (%)	6 (21.4)
LVAD-related infection, n (%)	17 (60.7)

BTT: bridge to transplant; GI: gastrointestinal; LVAD: left ventricular assist device.

**Table 3:** Operative data and early outcomes after heart transplantation.

	BTT (n = 28)	DTT (n = 77)	*p* Value
CPB time, minutes	217.7 ± 58.3	146.2 ± 53.3	0.001
CC time, minutes	113.9 ± 29.4	79.3 ± 15.6	0.001
Bleeding requiring SR	4 (14.3)	7 (9.1)	0.442
Cerebrovascular events, n (%)	3 (10.7)	1 (1.3)	0.057
Hemodialysis, n (%)	13 (46.4)	39 (50.6)	0.702
Acute rejection, n (%)	2 (7.1)	9 (11.7)	0.723
ECMO, n (%)	7 (25.0)	22 (28.6)	0.717
Length of hospital stay, days	23 (20–28)	22 (20–27)	0.875
Hospital mortality, n (%)	1 (3.6)	4 (5.2)	0.999

BTT: bridge to transplant; CC: cross-clamping; CPB: cardiopulmonary bypass; DTT: direct-to-transplant; ECMO: Extra Corporeal Membrane Oxygenation.

**Table 4:** Secondary outcomes at last follow-up.

	BTT (n = 28)	DTT (n = 77)	*p* Value
Permanent AF/PM/ICD	3 (10.7)	8 (10.4)	0.999
Cerebrovascular events *	3 (10.7)	2 (2.6)	0.117
CAV	4 (14.2)	14 (18.2)	0.775
Grade I	2	10	
Grade II	0	3	
Grade III	2	1	
eGFR < 30 mL/min	4 (14.2)	18 (23.4)	0.420
Permanent dialysis	1 (3.6)	11 (14.3)	0.175
Organ rejection	21 (75.0)	61 (79.2)	0.644
Grade 1R	16	57	
Grade 2R	4	3	
Grade 3R	1	1	

AF: atrial fibrillation; BTT: bridge to transplant; CC: cross-clamping; CAV: cardiac allograft vasculopathy; DTT: direct-to-transplant; eGFR: estimated Glomerular Filtration Rate; ICD: Implantable Cardioverter-Defibrillator; PM: pacemaker; * previous cerebrovascular events were excluded.

## Data Availability

The raw data supporting the conclusions of this article will be made available by the authors on request.
